# MALE SPOUSAL CAREGIVERS ARE ON THE RISE BUT THEY PROVIDE MOSTLY SECONDARY, INSTRUMENTAL, AND LOW-INTENSITY CARE

**DOI:** 10.1093/geroni/igae098.3152

**Published:** 2024-12-31

**Authors:** Hyungmin Cha, Jennifer Ailshire

**Affiliations:** University of Southern California, Los Angeles, California, United States; University of Southern California, Los Angeles, California, United States

## Abstract

Despite demographic projections that more men are adopting caregiving roles than ever before, there is a lack of population-level estimates regarding the prevalence of male caregivers over time. It is also unclear which types of caregiving tasks men typically provide. Using data from the Health and Retirement Study (2002-2018), we investigate trends in the prevalence of spousal caregiving among both men and women. We also analyze subcategories of caregiving, including primary versus secondary caregiving, activities of daily living versus instrumental activities of daily living, and high-intensity versus low-intensity care. Our results indicate that the prevalence of male caregivers has been increasing from 2002 to 2018, but these caregivers primarily focus on secondary, instrumental, and low-intensity caregiving. Further descriptive statistics comparing male caregivers in 2002 to those in 2018 reveal that this trend may be attributed to a growing number of Hispanic individuals, younger cohorts, highly educated, retired, and wealthy men, as well as those with older spouses. Our findings reveal the increase in male caregivers over time, which reshapes the gendered profile of caregivers. Nevertheless, male caregivers still primarily provide secondary, instrumental, and low-intensity care.

